# Supplementary material for: LEDGF/p75 Is Required for an Efficient DNA Damage Response
Source: Int J Mol Sci. 2021 May 30;22(11):5866. doi: 10.3390/ijms22115866 (PMC8198318; doi:10.3390/ijms22115866)
Supplement: Supplementary file 1 [file ijms-22-05866-s001.zip › ijms-1207674-supplementary.pdf]

A.

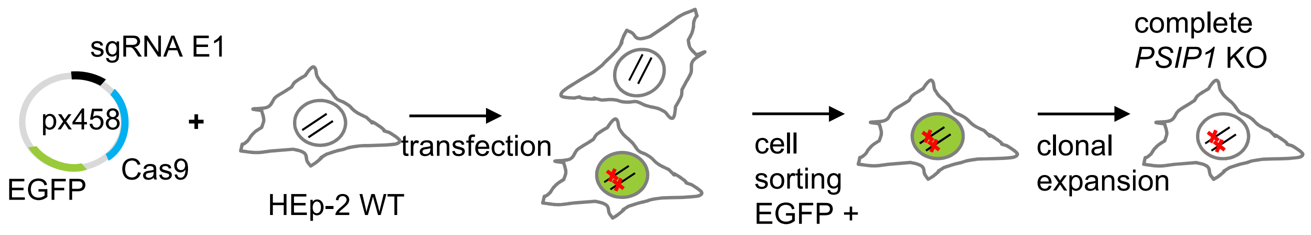

B.

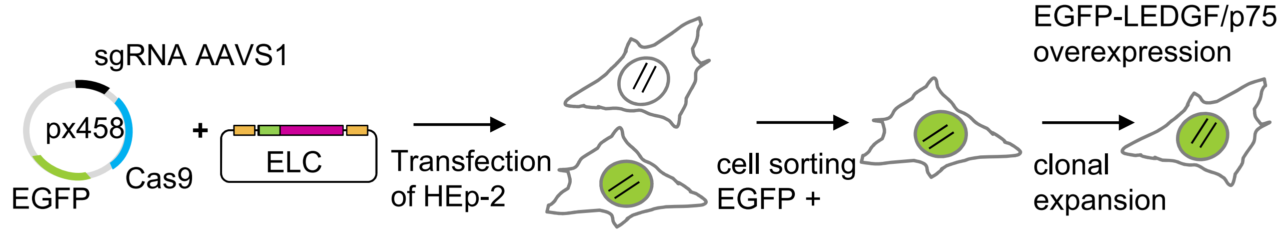

C.

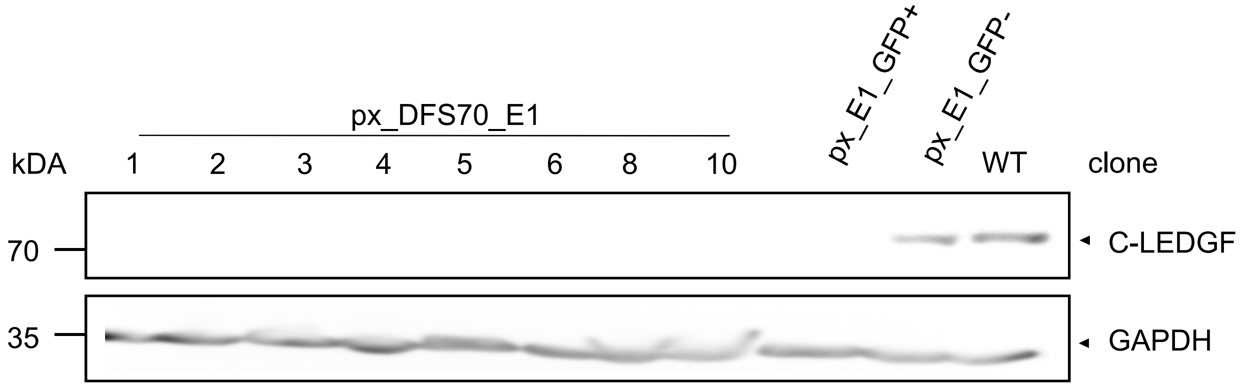

D.

Clone	Selection marker	Mutations	Premature stop codon (pos. in CDS)
**LEDGF KO**	EGFP	32 nt del<br>11 nt del	aa 19 (exon 2)<br>aa 26 (exon 2)

E.

Gene locus	Chromosome	Area	Target sequence	Mutation
**MTSS1L**	16	Intronic	AGAT**T**AAAGG**C**T**C**TCCCCAT	-
**SLC9C1**	13	Intronic	A**A**A**A**GAAA**T**GTTATCCCCAT	-
**TUB**	11	Exonic	AGA**G**GAA**G**GGT**G**A**G**CCCCAT	-

Figure S1: Verification of LEDGF-modified HEp-2 cells.

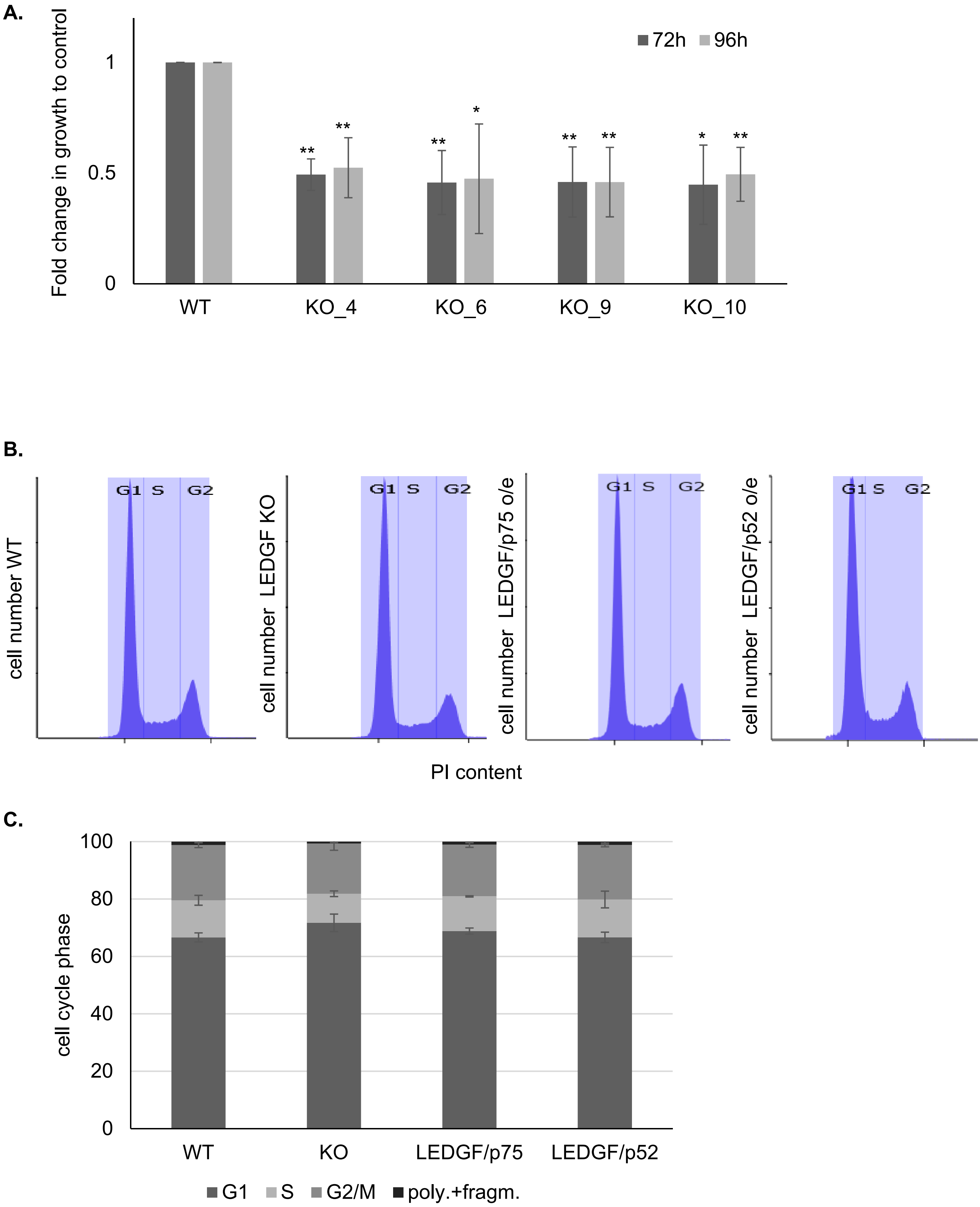

Figure S2: Proliferation rate and cell cycle analysis of LEDGF-modified cells.

A.

Clone	Selection marker	Mutations	Premature stop codon (pos. in CDS)
**PSIP1 KO**	EGFP	195 nt del + 5 nt insertion	Loss of exon 1

B.

Gene locus	Chromosome	Area	Target sequence	Mutation
**MTSS1L**	16	Intronic	AGAT**T**AAAGG**C**T**C**TCCCCAT	-
**SLC9C1**	13	Intronic	A**A**A**A**GAAA**T**GTTATCCCCAT	-
**TUB**	11	Exonic	AGA**G**GAA**G**GGT**G**A**G**CCCCAT	-

C.

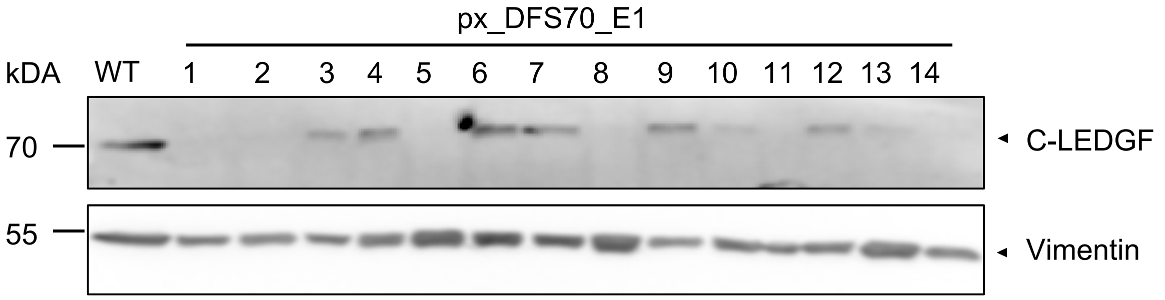

D.

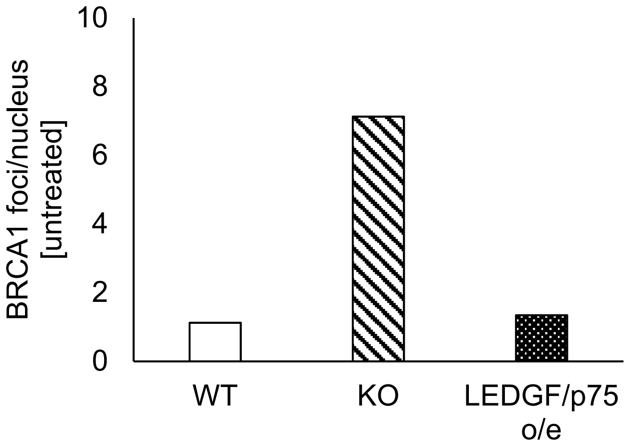

E.

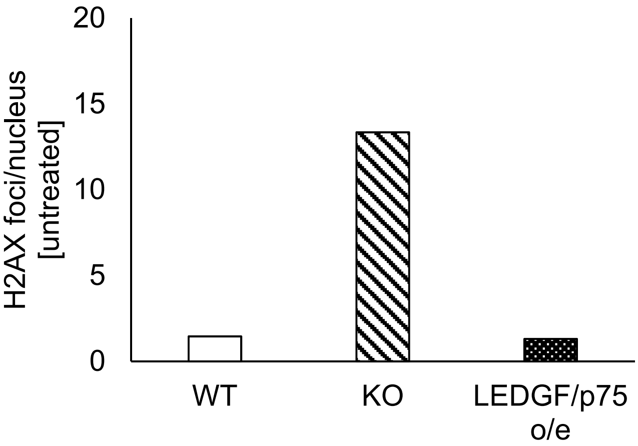

Figure S3: Verification of U2OS modified cells

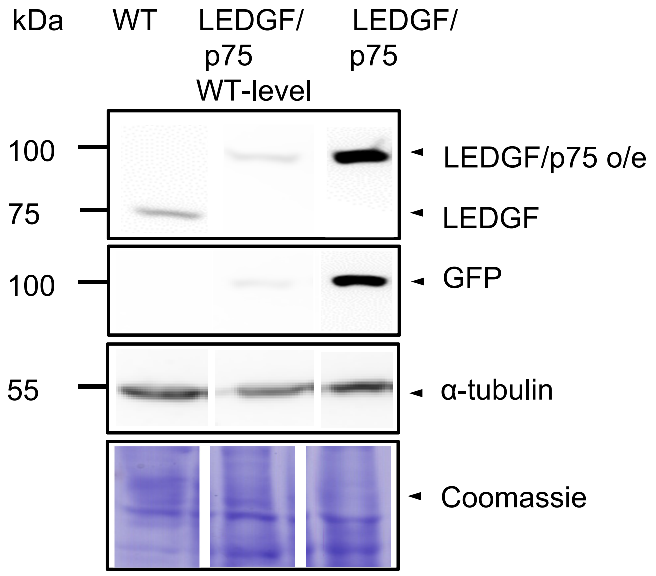

Figure S4: C. Different LEDGF expression levels of LEGF/p75 re-expression clones.

attB1	ACAAGTTTGTACAAAAAAGCAGGCT
EGFP	ATGGTGAGCAAGGGCGAGGAGCTGTTCACCGGGGTGGTGCCCATCC TGGTCGAGCTGGACGGCGACGTAAACGGCCACAAGTTCAGCGTGTC CGGCGAGGGCGAGGGCGATGCCACCTACGGCAAGCTGACCCTGAAG TTCATCTGCACCACCGGCAAGCTGCCCGTGCCCTGGCCCACCCTCGT GACCACCCTGACCTACGGCGTGCAGTGCTTCAGCCGCTACCCCGACC ACATGAAGCAGCACGACTTCTTCAAGTCCGCCATGCCCGAAGGCTAC GTCCAGGAGCGCACCATCTTCTTCAAGGACGACGGCAACTACAAGAC CCGCGCCGAGGTGAAGTTCGAGGGCGACACCCTGGTGAACCGCATC GAGCTGAAGGGCATCGACTTCAAGGAGGACGGCAACATCCTGGGGC ACAAGCTGGAGTACAACTACAACAGCCACAACGTCTATATCATGGCCG ACAAGCAGAAGAACGGCATCAAGGTGAACTTCAAGATCCGCCACAAC ATCGAGGACGGCAGCGTGCAGCTCGCCGACCACTACCAGCAGAACA CCCCCATCGGCGACGGCCCCGTGCTGCTGCCCGACAACCACTACCT GAGCACCCAGTCCGCCCTGAGCAAAGACCCCAACGAGAAGCGCGAT CACATGGTCCTGCTGGAGTTCGTGACCGCCGCCGGGATCACTCTCGG CATGGACGAGCTGTACAAG
LEDGF/p75	ACTCGCGATTTCAAACCTGGAGACCTCATCTTCGCCAAGATGAAAGGT TATCCCCATTGGCCAGCTCGAGTAGACGAAGTTCCTGATGGAGCTGT AAAGCCACCCACAAACAAACTACCCATTTTCTTTTTTGGAACTCATGAG ACTGCTTTTTTAGGACCAAAGGATATATTTCCTTACTCAGAAAATAAGG AAAAGTATGGCAAACCAAATAAAAGAAAAGGTTTTAATGAAGGTTTATG GGAGATAGATAACAATCCAAAAGTGAAATTTTCAAGTCAACAGGCAGC AACTAAACAATCAAATGCATCATCTGATGTTGAAGTTGAAGAAAAGGAA ACTAGTGTTTCAAAGGAAGATACCGACCATGAAGAAAAAGCCAGCAAT GAGGATGTGACTAAAGCAGTTGACATAACTACTCCAAAAGCTGCCAGA AGGGGGAGAAAGAGAAAGGCAGAAAAACAAGTAGAAACTGAGGAGGC AGGAGTAGTGACAACAGCAACAGCATCTGTTAATCTAAAAGTGAGTCC TAAAAGAGGACGACCTGCAGCTACAGAAGTCAAGATTCCAAAACCAAG AGGCAGACCCAAAATGGTAAAACAGCCCTGTCCTTCAGAGAGTGACA TCATTACTGAAGAGGACAAAAGTAAGAAAAAGGGGCAAGAGGAAAAAC AACCTAAAAAGCAGCCTAAGAAGGATGAAGAGGGCCAGAAGGAAGAA GATAAGCCAAGAAAAGAGCCGGATAAAAAAGAGGGGAAGAAAGAAGT TGAATCAAAAAGGAAAAATTTAGCTAAAACAGGGGTTACTTCAACCTC CGATTCTGAAGAAGAAGGAGATGATCAAGAAGGTGAAAAGAAGAGAA AAGGTGGGAGGAACTTTCAGACTGCTCACAGAAGGAATATGCTGAAA GGCCAACATGAGAAAGAAGCAGCAGATCGAAAACGCAAGCAAGAGGA ACAAATGGAAACTGAGCAGCAGAATAAAGATGAAGGAAAGAAGCCAG AAGTTAAGAAAGTGGAGAAGAAGCGAGAAACATCAATGGATTCTCGAC TTCAAAGGATACATGCTGAGATTAAAAATTCACTCAAAATTGATAATCT TGATGTGAACAGATGCATTGAGGCCTTGGATGAACTTGCTTCACTTCA GGTCACAATGCAACAAGCTCAGAAACACACAGAGATGATTACTACACT GAAAAAAATACGGCGATTCAAAGTTAGTCAGGTAATCATGGAAAAGTC TACAATGTTGTATAACAAGTTTAAGAACATGTTCTTGGTTGGTGAAGGA GATTCCGTGATCACCCAAGTGCTGAATAAATCTCTTGCTGAACAAAGA CAGCATGAGGAAGCGAATAAAACCAAAGATCAAGGGAAGAAAGGGCC AAACAAAAAGCTAGAGAAGGAACAAACAGGGTCAAAGACTCTAAATGG AGGATCTGATGCTCAAGATGGTAATCAGCCACAACATAACGGGGAGA GCAATGAAGACAGCAAAGACAACCATGAAGCCAGCACGAAGAAAAAG CCATCCAGTGAAGAGAGAGAGACTGAAATATCTCTGAAGGATTCTACA CTAGATAAC
attB2	ACCCAGCTTTCTTGTACAAAGTGGT

Figure S5: HDR template for EGFP-LEDGF/p75 overexpression in human AAVS1 locus.

attB1	ACAAGTTTGTACAAAAAAGCAGGCT
mEmarald	ATGGTGAGCAAGGGCGAGGAGCTGTTCACCGGGGTGGTGCCCATCC<br>TGGTCGAGCTGGACGGCGACGTAAACGGCCACAAGTTCAGCGTGTC<br>CGGCGAGGGCGAGGGCGATGCCACCTACGGCAAGCTGACCCTGAAG<br>TTCATCTGCACCACCGGCAAGCTGCCCGTGCCCTGGCCCACCCTCGT<br>GACCACCTTGACCTACGGCGTGCAGTGCTTCGCCCGCTACCCCGACC<br>ACATGAAGCAGCACGACTTCTTCAAGTCCGCCATGCCCGAAGGCTAC<br>GTCCAGGAGCGCACCATCTTCTTCAAGGACGACGGCAACTACAAGAC<br>CCGCGCCGAGGTGAAGTTCGAGGGCGACACCCTGGTGAACCGCATC<br>GAGCTGAAGGGCATCGACTTCAAGGAGGACGGCAACATCCTGGGGC<br>ACAAGCTGGAGTACAACTACAACAGCCACAAGGTCTATATCACCGCC<br>GACAAGCAGAAGAACGGCATCAAGGTGAACTTCAAGACCCGCCACAA<br>CATCGAGGACGGCAGCGTGCAGCTCGCCGACCACTACCAGCAGAAC<br>ACCCCCATCGGCGACGGCCCCGTGCTGCTGCCCGACAACCACTACC<br>TGAGCACCCAGTCCAAGCTGAGCAAAGACCCCAACGAGAAGCGCGAT<br>CACATGGTCCTGCTGGAGTTCGTGACCGCCGCCGGGATCACTCTCGG<br>CATGGACGAGCTGTACAAG
LEDGF/p75	ATGACTCGCGATTTCAAACCTGGAGACCTCATCTTCGCCAAGATGAAA<br>GGTTATCCCCATTGGCCAGCTCGAGTAGACGAAGTTCCTGATGGAGC<br>TGTAAAGCCACCCACAAACAAACTACCCATTTTCTTTTTTGGAACTCAT<br>GAGACTGCTTTTTTAGGACCAAAGGATATATTTCCTTACTCAGAAAATA<br>AGGAAAAGTATGGCAAACCAAATAAAAGAAAAGGTTTTAATGAAGGTTT<br>ATGGGAGATAGATAACAATCCAAAAGTGAAATTTTCAAGTCAACAGGC<br>AGCAACTAAACAATCAAATGCATCATCTGATGTTGAAGTTGAAGAAAA<br>GGAAACTAGTGTTTCAAAGGAAGATACCGACCATGAAGAAAAAGCCAG<br>CAATGAGGATGTGACTAAAGCAGTTGACATAACTACTCCAAAAGCTGC<br>CAGAAGGGGGAGAAAGAGAAAGGCAGAAAAACAAGTAGAAACTGAGG<br>AGGCAGGAGTAGTGACAACAGCAACAGCATCTGTTAATCTAAAAGTGA<br>GTCCTAAAAGAGGACGACCTGCAGCTACAGAAGTCAAGATTCCAAAAC<br>CAAGAGGCAGACCCAAAATGGTAAAACAGCCCTGTCCTTCAGAGAGT<br>GACATCATTACTGAAGAGGACAAAAGTAAGAAAAAGGGGCAAGAGGA<br>AAAACAACCTAAAAAGCAGCCTAAGAAGGATGAAGAGGGCCAGAAGG<br>AAGAAGATAAGCCAAGAAAAGAGCCGGATAAAAAAGAGGGGAAGAAA<br>GAAGTTGAATCAAAAAGGAAAAATTTAGCTAAAACAGGGGTTACTTCA<br>ACCTCCGATTCTGAAGAAGAAGGAGATGATCAAGAAGGTGAAAAGAA<br>GAGAAAAGGTGGGAGGAACTTTCAGACTGCTCACAGAAGGAATATGC<br>TGAAAGGCCAACATGAGAAAGAAGCAGCAGATCGAAAACGCAAGCAA<br>GAGGAACAAATGGAAACTGAGCA
attB2	ACCCAGCTTTCTTGTACAAAGTGGT

Figure S6: HDR template for mEmarald_LEDGF/p52 overexpression in human AAVS1 locus.

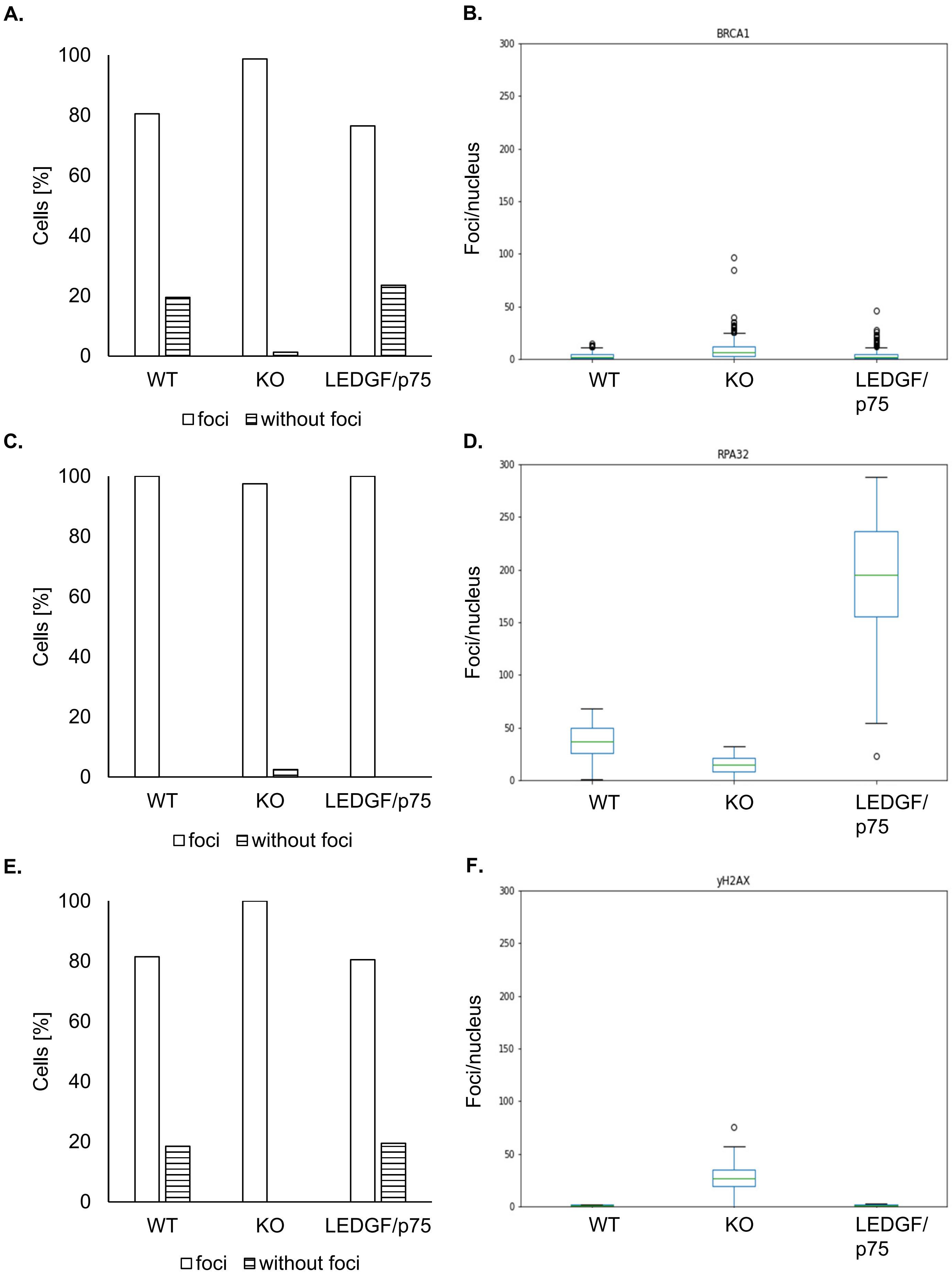

Figure S7: Nuclear foci analysis of HEp-2 cells
